# Development and accuracy validation of a fat fraction imaging biomarker for sialadenitis in the parotid gland

**DOI:** 10.1186/s12903-023-03024-9

**Published:** 2023-06-01

**Authors:** Ari Lee, Yoon Joo Choi, Kug Jin Jeon, Sang-Sun Han, Chena Lee

**Affiliations:** Department of Oral and Maxillofacial Radiology, College of Dentistry, Yonsei University, 50-1 Yonsei-ro Seodaemun-gu, Seoul, 03722 Korea

**Keywords:** Imaging biomarker, Magnetic resonance imaging, Sialadenitis, Salivary gland, Diagnostic imaging

## Abstract

**Background:**

The diagnosis of sialadenitis, the most frequent disease of the salivary glands, is challenging when the symptoms are mild. In such cases, biomarkers can be used as definitive diagnostic indicators. Recently, biomarkers have been developed by extracting and analyzing pathological and morphological features from medical imaging. This study aimed to establish a diagnostic reference for sialadenitis based on the quantitative magnetic resonance imaging (MRI) biomarker IDEAL-IQ and assess its accuracy.

**Methods:**

Patients with sialadenitis (n = 46) and control subjects (n = 90) that underwent MRI were selected. Considering that the IDEAL-IQ value is a sensitive fat fractional marker to the body mass index (BMI), all subjects were also categorized as under-, normal-, and overweight. The fat fraction of parotid gland in the control and sialadenitis groups were obtained using IDEAL-IQ map. The values from the subjects in the control and sialadenitis groups were compared in each BMI category. For comparison, t-tests and receiver operating characteristic (ROC) curve analyses were performed.

**Results:**

The IDEAL-IQ fat faction of the control and sialadenitis glands were 38.57% and 23.69%, respectively, and the differences were significant. The values were significantly lower in the sialadenitis group (P), regardless of the BMI types. The area under the ROC curve (AUC) was 0.83 (cut-off value: 28.72) in patients with sialadenitis. The AUC for under-, normal-, and overweight individuals were 0.78, 0.81, and 0.92, respectively.

**Conclusions:**

The fat fraction marker based on the IDEAL-IQ method was useful as an objective indicator for diagnosing sialadenitis. This marker would aid less-experienced clinicians in diagnosing sialadenitis.

## Background

Research on establishing image biomarkers by extracting and analyzing specific features from medical imaging has been actively conducted recently [1–4]. Imaging biomarkers have been developed as indicators for diagnosing diseases and predicting prognoses by extracting and analyzing pathological and morphological features from medical images [3]. These image-based biomarkers may quantitatively detect and identify pathology, compared with the previously adopted procedures that depended on subjective radiologist decisions [4, 5].

In particular, magnetic resonance imaging (MRI) is a sensitive imaging modality explaining the specific properties of organ tissues. Several studies have reported the development of MRI-based biomarkers and their application in predicting the prognosis of disease or cancer staging [1–4, 6–8]. A recently proposed fat quantification method is the iterative decomposition of water and fat with echo asymmetry and least-squares estimation (IDEAL-IQ). This method has been introduced as an accurate and reproducible tool for fat measurements [9]. IDEAL-IQ separates water and fat signals using multi-echo chemical shifts and can more accurately quantify fat (which comprises multiple spectral peaks) [10].

Several salivary gland fat fraction studies based on this technique have been reported [11–13, 25]. These studies have reported the fat contents of major salivary glands of healthy people and concluded that the fat fraction of the gland increases with age and body mass, even in normal salivary glands [11–13]. In particular, the body mass index (BMI) was highly correlated with age; however, sex was not correlated with gland fat fraction [11–13]. These studies confirmed that the fat fraction differs among individuals in the healthy population owing to physiological factor differences. However, the critical quantitative values that can aid the diagnosis of salivary gland disease according to physiological factors have not been presented. Therefore, based on these studies, a comparative evaluation with the values obtained from diseased salivary glands is necessary.

Inflammatory diseases are representative of salivary gland pathology and have the highest frequency among salivary gland diseases [14]. In particular, they can occur in all age groups; yet, they frequently affect older people with reduced salivary gland function, patients who have undergone radiation therapy, and those with immunocompromised status [15]. Depending on the disease progression state, the inflammatory lesion of the salivary gland causes histological changes and shows various signal intensity alterations in MRI compared with other normal glands. Diagnosis of sialadenitis is vital in initiating conservative treatment [16, 26, 27]. Diagnosis can be challenging if the clinical symptoms are mild and the imaging signal is ambiguous. Based on the accurate quantitative imaging technique of IDEAL-IQ, the fat fraction would be a helpful diagnostic marker, especially for clinicians with less clinical experience or difficulty in recognizing minor signal changes in images.

Therefore, this study aimed to establish a diagnostic reference value of sialadenitis using the IDEAL-IQ method of MRI and assess its diagnostic accuracy. Additionally, the diagnostic reference was established according to BMI by considering that IDEAL-IQ is a BMI-sensitive marker.

## Methods

This study was approved by the Institutional Review Board of Yonsei University Dental Hospital (No. 2-2021-0058) and was conducted and completed in accordance with relevant guidelines and ethical regulations. Owing to the retrospective nature of this study, the need for informed consent was waived by the Institutional Review Board of Yonsei University Dental Hospital.

### Study population

The records of patients who underwent MRI examinations from July to December 2020 were reviewed. Patients with clinical and imaging diagnoses of sialadenitis were included in the sialadenitis group. Patients who did not have symptoms or any previous medical history related to salivary gland disease were included in the control group. Patients with salivary gland related disease (neoplasm or autoimmune disease) other than sialadenitis were all excluded. Patients who had symptoms of dry mouth or massive medication status were also excluded based on a thorough clinical record review.

In control individuals, either the right or left side of the glands were randomly chosen using the “sample” function in R studio (R Foundation for Statistical Computing, Vienna, Austria). The general information of the subjects, including age, sex, BMI, body weight, and height, were thoroughly reviewed. The subjects were classified into three BMI groups: underweight (BMI < 18.50 kg/m^2^), normal weight (18.50 < BMI < 24.99 kg/m^2^), and overweight (BMI ≥ 25.00 kg/m^2^) according to the World Health Organization criteria [17].

### Scan parameters

All MRI scans were acquired using a 3.0 T scanner (Pioneer, GE Healthcare, Waukesha, WI, USA) with a 16-channel flex reception coil. IDEAL-IQ and transverse relaxation T2-weighted images in axial views were included in this study. The imaging parameters of IDEAL-IQ were as follows: echo time: 1.00, 2.04, 3.08, 4.12, 5.16, 6.20 ms; echo train length, 3; repetition time, 10.52–10.63 ms; bandwidth, 868.047 kHz; number of excitations, 1.0; field-of-view, 240 × 240 mm; slice thickness, 4.0 mm; scan time, 1 min; flip angle, 4°; and matrix, 160 × 160. The imaging parameters of the T2-weighted images were as follows: echo time, 85 ms; echo train length, 9; repetition time, 3100 ms; bandwidth, 83.33 kHz; number of excitations, 1.0; field-of-view, 230 × 230 mm; slice thickness, 4.0 mm; scan time, 2:17 min; flip angle, 111; and matrix, 380 × 320.

### MR imaging analysis

**Fig. 1:**
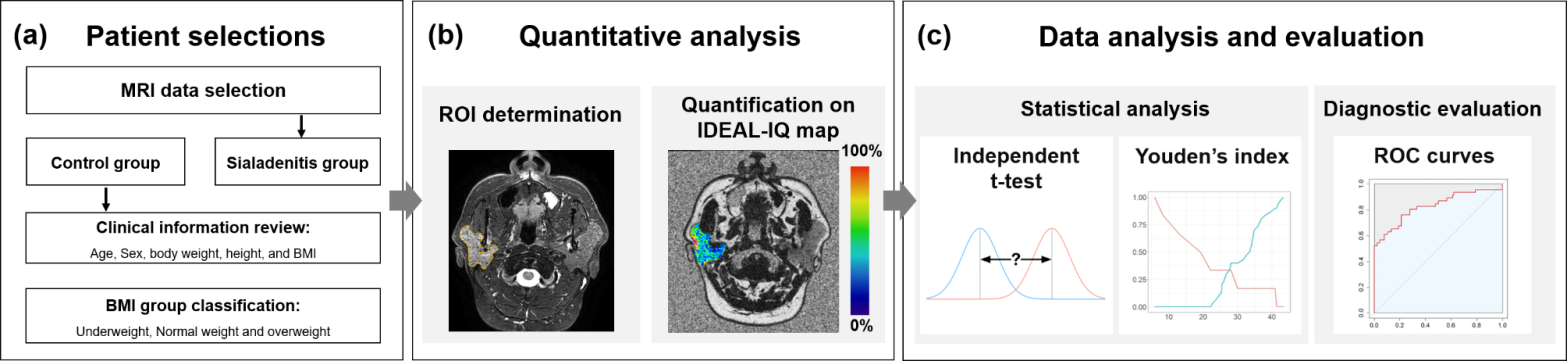
Flowchart of the study process to establish diagnostic reference values. **(a)** Patient selections. **(b)** Quantitative analysis. **(c)** Data analysis and evaluation

The imaging analysis was performed on IDEAL-IQ (Fig. 1a). Regions of interest (ROIs) were drawn along the border of the parotid gland in the axial view of the IDEAL-IQ, where the glands were most visible. The T2-weighted image was referenced for ROI determination (Fig. 1b). The IDEAL-IQ fat fraction within the ROI was obtained. Two observers performed the same measurement, and the first observer conducted the measurement twice with an interval of 1 month for inter- and intra-observer reliability analysis. The first observer was a graduate student majoring in oral radiology, and the second observer was an oral radiologist with more than 15 years of experience.

### Statistical analyses

The mean and standard deviation were obtained from the imaging measured value. The normal and inflamed gland groups were compared using an independent t-test (Fig. 1c). To differentiate normal glands from sialadenitis according to the BMI, receiver operating characteristic (ROC) curves analysis was performed for IDEAL-IQ based-fat fraction (%). Optimal cut-off points were selected according to Youden’s index. Sensitivity and specificity values were used to evaluate the accuracy of the analyzed results. The intra-class correlation coefficients of the measurement values were evaluated with 95% confidence intervals (CI). Statistical analyses were conducted in R Studio (version 3.5.3).

## Results

**Fig. 2:**
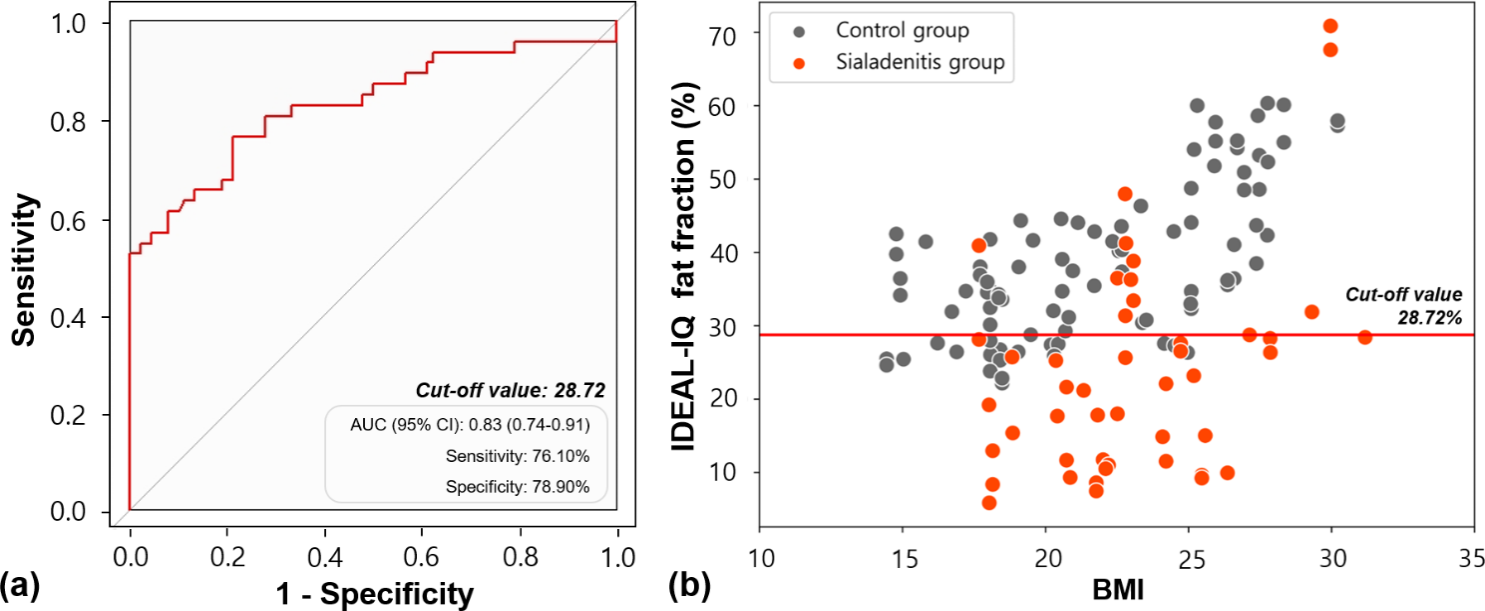
**(a)** Receiver operating characteristic (ROC) curve comparing the diagnostic performance of the percentage variation of fat fraction in its capacity to differentiate normal and inflamed glands. **(b)** Scatter plot demonstrating the distribution of IDEAL-IQ in the control and sialadenitis groups. The red line indicates the cut-off value

The clinical information of the study population is described in Table 1. The IDEAL-IQ based-fat fraction obtained from the sialadenitis group was significantly lower than that in the control group (p < 0.001) (Table 2). Regardless of the BMI, the difference between the sialadenitis and control groups is described in Table 2, and the difference was significant for the underweight (p < 0.001), normal (p < 0.001), and overweight (p < 0.001) groups. The area under the ROC curve (AUC) of the control and sialadenitis groups was 0.83 (95% CI, 0.74–0.91) (Fig. 2a), with statistical significance (p < 0.001). According to the BMI, the AUCs for sialadenitis (compared with normal glands) were 0.78, 0.81, and 0.92, respectively, in under-, normal-, and overweight patients (Table 3).

**Table 1:** Clinical information of the studied subjects

	Control group (n = 90)	Sialadenitis group (n = 46)
**Age** (year)	40.51 ± 16.76	38.65 ± 14.85
**Sex** (N)		
Male	33	34
Female	57	12
**BMI** (kg/m^2^)	21.84 ± 4.27	23.05 ± 3.46
**BMI group** (N)		
Underweight	30	19
Normal weight	30	6
Overweight	30	21

Abbreviation: BMI, body mass index

**Table 2:** Comparison of IDEAL-IQ fat fraction (%) of patients in the control and sialadenitis groups

	Control group	Sialadenitis group	P-value
Total	38.57 ± 10.33	23.69 ± 14.41	< 0.001*
Underweight	31.69 ± 6.02	19.20 ± 13.31	< 0.001*
Normal weight	35.46 ± 6.82	21.48 ± 11.60	< 0.001*
Overweight	48.55 ± 9.11	27.54 ± 17.09	< 0.001*

Mean ± standard deviation (%)

*p < 0.001, independent t-test

**Table 3:** AUC, SE, 95% confidence interval, and p-values of the three BMI control and sialadenitis groups

	AUC	SE	95% confidence interval	
			Lower	Upper
Underweight	0.78	0.03	0.51	1.00
Normal weight	0.81	0.05	0.72	0.91
Overweight	0.92	0.03	0.86	0.99

Abbreviations: AUC, area under the curve; SE, standard error; BMI, body mass index

Using Youden’s index, the IDEAL-IQ cut-off value for sialadenitis glands was 28.72% (sensitivity, 76.10%; specificity, 78.90%) (Fig. 2b). For individual BMI groups, the cut-off values for under-, normal-, and overweight patients were 19.17%, 25.77%, and 32.06%, respectively (Table 4). The intra- and inter-observer ICCs were 0.994 (95% CI, 0.968–0.992) and 0.953 (95% CI, 0.907–0.976).

**Table 4:** Cut-off value of sialadenitis using IDEAL-IQ fat fraction according to BMI groups

	Cut-off value (%)	Sensitivity	Specificity
Underweight	19.17	66.70%	100%
Normal weight	25.77	60.90%	100%
Overweight	32.06	80.40%	100%

Abbreviation: BMI, body mass index

## Discussion

Despite the recent development of various imaging biomarkers, studies conducted on the salivary gland as a target organ are very rare. IDEAL-IQ has been known as an objective MR-based imaging biomarker for identifying unique tissue characteristics [18, 19]. This method has been reported to show high accuracy and reproducibility for quantitative diagnosis [9]. In this study, we targeted inflammatory disease of the parotid gland due to its high incidence among salivary gland diseases, which led to a large sample size. Thus, using the IDEAL-IQ technique, we found that the diagnostic accuracy of the respective imaging biomarker was adequate to distinguish between normal salivary glands and sialadenitis in parotid glands. In addition, the fat fraction value acquired with the IDEAL-IQ method presented precise cut-off values suitable for all three BMI groups.

Currently, IDEAL-IQ is extensively used for quantitative diagnosis of hepatic steatosis in clinics [18, 19]. Although there have been studies attempting to analyze the salivary glands based on this technique, they are confined to establishing fat fraction values of the normal gland, and the pathologic diagnostic model was not feasible [11, 14]. In this study, we compared the fat fractions between the diseased and normal groups. Significant results were obtained, in which the value was low in the salivary glands diagnosed with chronic, acute, or acute chronic inflammation (23.69%<35.57%; p < 0.05). This is thought to be attributed to the fact that the adipose tissue, which normally occupies the parenchyma of the salivary gland, is filled with edematous fluid in acute sialadenitis [20] and degenerates into fibrosis in chronic sialadenitis cases [20, 21]. This result was comparable to that obtained by Chikui et al. in 2018 [22]. They compared the fat fraction value of three different salivary gland diseases, namely, Sjögren’s syndrome, immunoglobulin G4-related dacryoadenitis, and sialadenitis, with that of normal glands [22]. They also reported significantly lower fat fractions and lower values in sialadenitis than those in normal salivary glands.

In normal glands, the fat fraction value obtained using IDEAL-IQ in the study by Chikui et al. was comparable to that of the current study [22]. However, the value was quite different in the sialadenitis group. The previous study conducted measurements in 46 normal glands and six sialadenitis parotid gland cases [22]. In contrast, the current study included a large number of subjects (90 normal and 46 sialadenitis). Thus, the variance of age and BMI distribution had considerable effects on the differences in the fat fraction between the previous and our study. Furthermore, as mentioned in previous literature, there may be differences depending on the MRI instrument [23]. Schneider et al. concluded that IDEAL-IQ showed vendor-dependent differences in the range of 2.4–3.8% in fat fraction measurement. Taking this into consideration, the differences in the measured values in normal glands, which is approximately equal to 1%, can be considered a result of the vendor difference.

A significant finding of our study was the precise diagnostic accuracy in the overweight group with high BMI. It is challenging to distinguish pathologic fatty degeneration from normal status in overweight patients. MRI fat signal is ambiguous in differentiating the pathologic change from that of the high fat content of the gland by overweight of general body mass in MRI. According to the results of this study, the IDEAL-IQ method showed that the AUC was in the range of 0.78–0.80 in the underweight and normal BMI groups (which was relatively accurate) and 0.9 in the overweight group, which can be considered an excellent accuracy outcome. However, the sensitivity was in the range of 66–80%, and the specificity reached 100%. Thus, it can be concluded that this marker yielded better performance in terms of its true-negative rate. Based on the value obtained from the IDEAL-IQ method, the diagnostic ability of the normal gland as normal (true-negative rate) was superior to the diagnosis of actual sialadenitis as sialadenitis (true-positive rate). A test with high specificity and with high true-negative rate is more suitable for a rule-in test [24]. The diagnostic errors related to low sensitivity can be corrected with a larger sample size. This is an area that requires attention in the clinical application of the IDEAL-IQ for diagnosing salivary gland disease.

Moreover, it can be assumed that the tissue characteristics of the salivary glands would be different even within the sialadenitis group depending on whether the disease is in the acute or chronic stage or the frequency of the disease occurrence. According to previous studies, the parenchyma of the salivary glands became fibrotic when chronic inflammation progressed while it did not present irreversible tissue degeneration in acute stages [20, 21].

This study had some limitations. First, it had a retrospective design for sample collection. Although the sample size of the current study was comparable to that of previous studies on parotid gland fat fraction, more realistic cut-off value for diagnosis could be expected when the study is conducted on a larger number of samples [11, 22]. Thus, multi-center large cohort studies are warranted to establish reliable IDEAL-IQ marker for clinical usage. Another weakness of this study was that the subjects included in the study exhibited variable disease progression stages with different frequencies and inflammatory disease depths. However, owing to the initial causative factor and the duration of the sialadenitis being ambiguous, we could not consider the stage of sialadenitis in the present study.

Despite the above limitations, the findings of the study are important as they propose a quantifiable diagnostic tool for salivary gland disease. In particular, quantitative diagnostic criteria were provided by the IDEAL-IQ technique. Considering that chronic or frequent acute sialadenitis may eventually lead to salivary gland dysfunction, the proposed biomarker may be considered an indicator for functional evaluations.

## Conclusions

In conclusion, the fat fraction value obtained using the IDEAL-IQ method was useful as an objective indicator when diagnosing parotid gland sialadenitis. Especially, this indicator would help less-experienced clinicians diagnose sialadenitis, which is currently ambiguous in imaging. In addition, when this method is used for diagnosing parotid gland sialadenitis, it would be appropriate to use different reference values according to the BMI.

## Data Availability

The datasets acquired during and/or analyzed during the current study are available from the corresponding author on reasonable request.

## Abbreviations

MRI: magnetic resonance imaging
BMI: body mass index
ROC: receiver operating characteristic
AUC: area under the ROC curve
IDEAL-IQ: iterative decomposition of water and fat with echo asymmetry and least-squares estimation
ROI: region of interest
